# Visualizing fusion of pseudotyped HIV-1 particles in real time by live cell microscopy

**DOI:** 10.1186/1742-4690-6-84

**Published:** 2009-09-18

**Authors:** Peter Koch, Marko Lampe, William J Godinez, Barbara Müller, Karl Rohr, Hans-Georg Kräusslich, Maik J Lehmann

**Affiliations:** 1 Department of Virology, Universitätsklinikum Heidelberg, Im Neuenheimer Feld 324, 69120 Heidelberg, Germany; 2 Department of Bioinformatics and Functional Genomics, BIOQUANT, IPMB, University of Heidelberg, Im Neuenheimer Feld 267, 69120 Heidelberg, Germany; 3 Division of Cell Biology, MRC Laboratory of Molecular Biology, Hills Road, Cambridge, CB20QH, UK

## Abstract

**Background:**

Most retroviruses enter their host cells by fusing the viral envelope with the plasma membrane. Although the protein machinery promoting fusion has been characterized extensively, the dynamics of the process are largely unknown.

**Results:**

We generated human immunodeficiency virus-1 (HIV-1) particles pseudotyped with the envelope (Env) protein of ecotropic murine leukemia virus eMLV to study retrovirus entry at the plasma membrane using live-cell microscopy. This Env protein mediates highly efficient pH independent fusion at the cell surface and can be functionally tagged with a fluorescent protein. To detect fusion events, double labeled particles carrying one fluorophor in Env and the other in the matrix (MA) domain of Gag were generated and characterized. Fusion events were defined as loss of Env signal after virus-cell contact. Single particle tracking of >20,000 individual traces in two color channels recorded 28 events of color separation, where particles lost the Env protein, with the MA layer remaining stable at least for a short period. Fourty-five events were detected where both colors were lost simultaneously. Importantly, the first type of event was never observed when particles were pseudotyped with a non-fusogenic Env.

**Conclusion:**

These results reveal rapid retroviral fusion at the plasma membrane and permit studies of the immediate post-fusion events.

## Background

Enveloped viruses enter host cells by membrane fusion at the plasma membrane or at intracellular membranes. This process is mediated by the interaction of cellular receptors and Env glycoproteins. Numerous studies have revealed detailed information about the proteins involved in fusion for many viruses and have elucidated fundamental principles of viral fusion mechanisms [1,2]. The dynamics of the fusion process, however, is still incompletely characterized. Furthermore, the early post-entry steps immediately following membrane fusion remain enigmatic for many viruses.

Previous investigations have employed bulk biochemical assays or cell-cell fusion to study the viral fusion process (for review see [3]). More recently, single particle tracking of fluorescently labeled viruses has become possible and has been successfully applied to characterize the entry of various viruses (for review see [4]). In most cases, the lipophilic dye DiD was used for labeling the membrane of enveloped virus particles [5-7]. As DiD is incorporated into the outer leaflet of the membrane its redistribution after virus-cell contact indicates primarily the lipid mixing of the contacting leaflets (termed hemifusion) and not the formation of the fusion pore [7].

HIV-1 entry, as well as entry of many other retroviruses, has long been believed to occur exclusively at the plasma membrane. More recently, however, productive infection by pH-independent, clathrin-dependent endocytosis of HIV-1 has also been reported [8] and was recently suggested to constitute the only route of productive entry [9]. We have developed a system to study the dynamics of HIV-1 entry based on fluorescent live cell microscopy, in which the MA domain of the main structural protein Gag is labeled by fusion to a fluorescent protein [10]. MA lines the inner surface of the viral membrane and is believed to separate from the core of the virion upon membrane fusion. The inner core is subsequently transformed into the reverse transcription complex, and after reverse transcription it is again transformed into the viral preintegration complex (PIC) (for review see [11]). These nucleoprotein complexes are poorly characterized, but are believed to contain no or only a small proportion of MA molecules [12]. MA is believed to remain at the site of fusion from where it is redistributed within the membrane or into the cytosol [13]. To allow for direct detection of fusion events, the fluorescent label at the MA domain was combined with a differently colored label at the core-associated viral protein R (Vpr), which remains associated with the PIC during cytoplasmic transport to the nucleus [14]. Fusion should thus be accompanied by a rapid separation of the two labels in this system. However, tracking >10,000 individual interactions at high time resolution did not yield clear separation events [15]. Since this may be due to the low fusogenicity of HIV, the possibility to pseudotype retroviruses was applied, and HIV-1 particles carrying the highly fusogenic glycoprotein of vesicular stomatitis virus (VSV-G) were analyzed. This approach resulted in readily detectable bulk color separation over time with the mRFP.Vpr that accumulated at the nuclear membrane and MA.eGFP exhibiting mostly cytoplasmic staining [15]. Thus, efficient fusion must have occurred, but only sporadic events of color separation were observed for individual particles. This raised the question as to whether membrane fusion may not be accompanied by immediate separation of the bulk of MA from the viral core. Furthermore, pseudotyping with VSV-G diverted the entry route of the particles to a pH dependent endocytic pathway, thereby potentially influencing subsequent events.

For these reasons we developed a system where the fate of the viral membrane can be unequivocally determined. We made use of fluorescent HIV particles, pseudotyped with an Env protein from eMLV. This approach provides two main advantages: First, MLV Env carrying particles targeting DFJ-8 cells with a high surface density of murine cationic aminoacid transporter (mCAT-1, the receptor for eMLV) represent one of the most efficient systems for studying pH independent fusion at the plasma membrane [16]. Second a well characterized fluorescent variant of eMLV Env is available which has been shown to mediate fusion with wild-type efficiency and remains associated with the host cell membrane after fusion [16]. We have studied the dynamics of retroviral fusion and investigated immediate post fusion events by live cell imaging using double labeled pseudotypes carrying the fluorescent variant of eMLV Env and the MA domain of HIV-1 Gag fused to another fluorescent protein. Here, we report single particle tracking of >20,000 individual traces of double-fluorescent pseudotyped HIV recording 28 events of color separation and 45 additional events, where both colors were lost simultaneously.

## Results

### Characterization of double labeled HIV-1 pseudotypes

**Figure 1:**
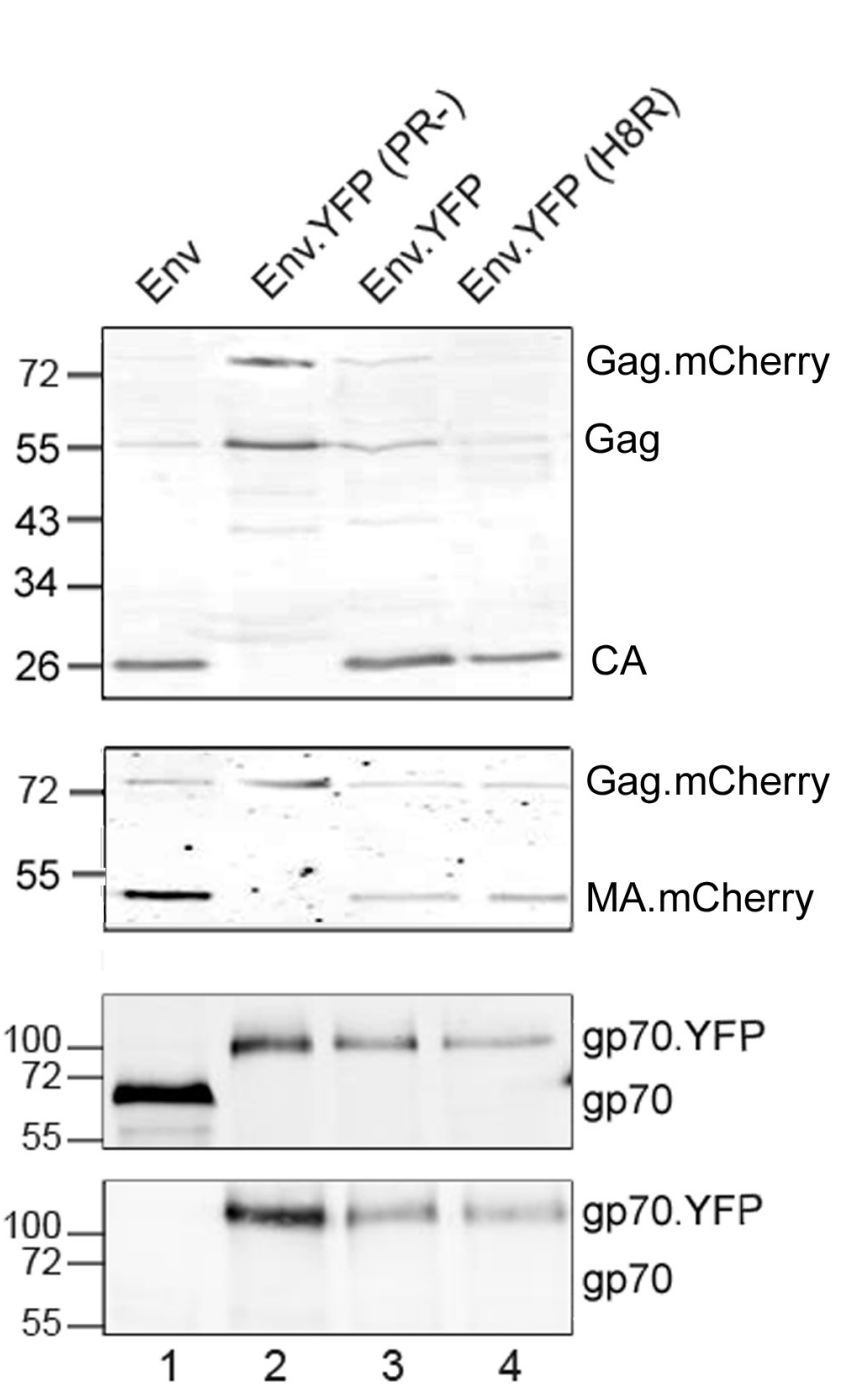
**Immunoblot analysis of purified particles**. pCHIV.mCherry derived particles pseudotyped with the indicated Env proteins were purified from the supernatant of 293T cells co-transfected with the respective plasmids by ultracentrifugation through a sucrose cushion. Samples were separated by SDS-PAGE (12.5% acrylamide), transferred to nitrocellulose according to standard procedures and proteins were detected by quantitative immunoblot (Li-Cor) using the following antisera: anti-CA (top panel); anti-mCherry (second panel); anti gp70 (third panel); anti-GFP (bottom panel). Positions of molecular mass standards (in kDa) are shown at the left.

To monitor the fusion of retroviral particles at the plasma membrane of living cells, we established a double labeling strategy in which a fluorescent label in the MA domain of HIV-1 Gag (MA.mCherry) was combined with another fluorescent label fused to eMLV Env (Env.YFP), which was then used to pseudotype HIV-1 particles. Both approaches have been described individually for functional labeling of viral particles [10,16], but had not been combined previously. Our initial aim was, therefore, to determine double labeling efficiency and its effects on viral infectivity. Previously, it was reported that an equimolar mixture of native and labeled HIV-1 Gag resulted in particles exhibiting wild-type infectivity, while particles made only from labeled Gag were significantly less infectious [10]. We therefore co-transfected 293T cells with an HIV-1 proviral plasmid lacking a functional *env *gene and its respective derivative carrying mCherry in the *gag *gene at an equimolar ratio and determined the optimal amount of co-transfected plasmid encoding Env.YFP by titration experiments. After sedimentation through a sucrose cushion, viral particles were immobilized on fibronectin-coated glass coverslips and imaged by epifluorescence microscopy to determine the degree of co-localization of the mCherry and YFP signals. Co-transfection of a two fold molar excess of Env.YFP encoding DNA resulted in at least 35% of all MA.mCherry carrying particles being detectably labeled also by Env.YFP (data not shown). Co-transfection of higher amounts of Env.YFP encoding plasmid affected the expression efficiency of the HIV derived plasmids, so that the production of particles was significantly reduced. The correct protein composition and the degree of Gag processing were confirmed for all particle preparations by immunoblotting using antisera against HIV-1 capsid (CA), MLV Env, and the fluorescent proteins mCherry and GFP, respectively (Figure 1).

### Analysis of Env-dependent fusion by fluorescence microscopy

**Figure 2:**
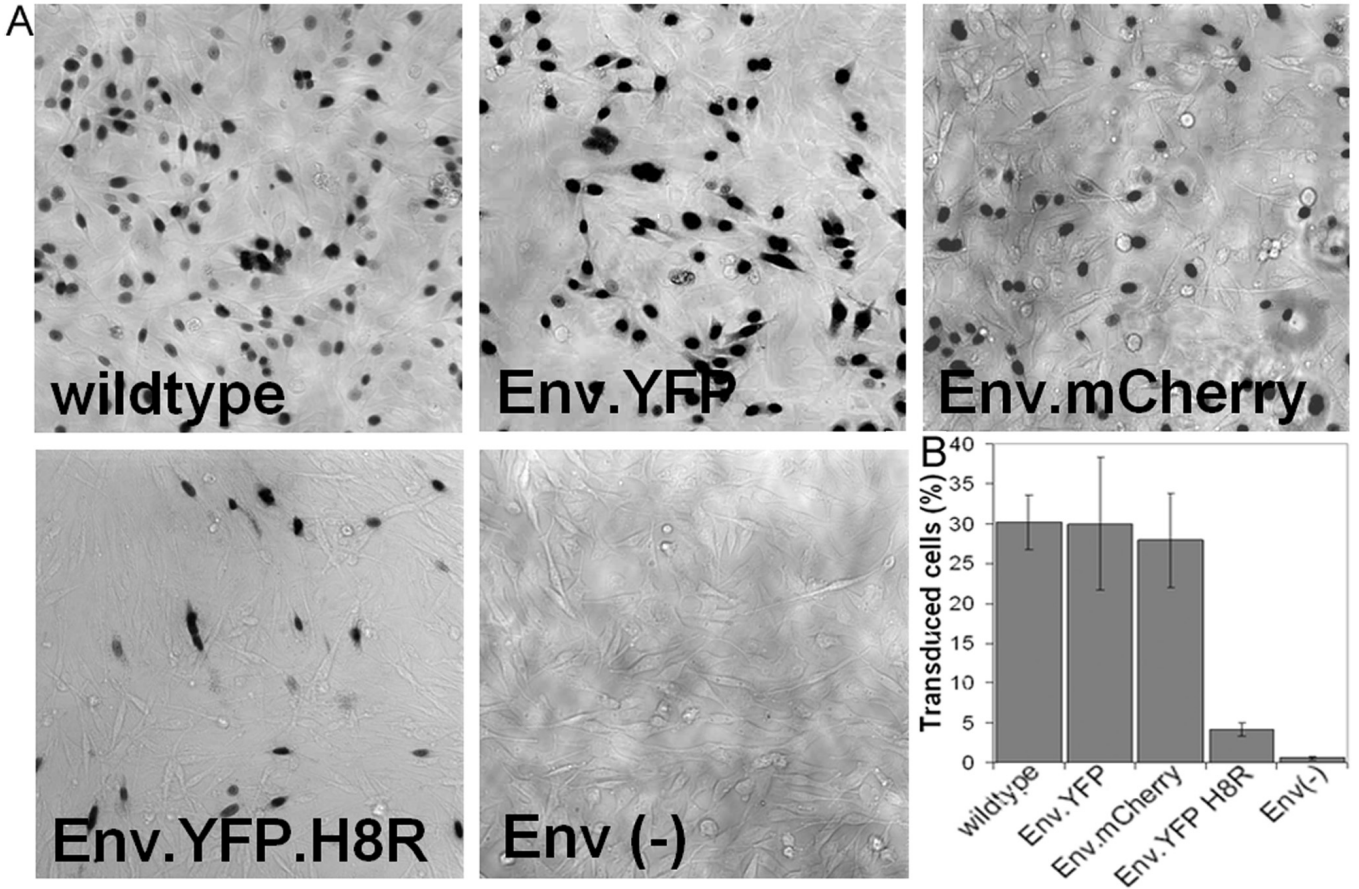
**Infectivity of glass-bound VLPs**. MLV-based vector particles carrying the β-galactosidase marker gene and the indicated Env proteins were purified from the supernatants of transfected 293T cells. Comparable amounts of particles (as determined by anti-MLV CA immunoblot) were adhered to fibronectin-coated coverslips, and DFJ-8 cells were allowed to settle on top of the VLP coated surface. (A) Following 48 hours of incubation at 37°C, cells were fixed and stained for β-galactosidase activity. (B) Infected cells were counted in 5 fields of view each (corresponding to ~500 cells) per experiment. The graph shows mean values and standard deviations from three independent experiments.

In order to visualize individual retroviral fusion events at the plasma membrane at high time resolution it is advantageous to maximize the number of productive virus-cell contacts occurring in the focal plane of the microscope. Thus, virus-cell interactions were monitored by epifluorescence microscopy after allowing cells to settle on top of a layer of particles bound to fibronectin coated chambered cover glasses rather than adding virus to adherent cells. This approach avoided displacement of cell surface associated viruses out of the microscopic focal plane due to cellular movement or membrane ruffling, which would lead to changes in signal intensities. Furthermore, this setup serves to synchronize the time of virus-cell contact. To determine whether virus particles that were immobilized on the glass surface retained infectivity, a β-galactosidase based infection assay was performed. To this end, equal amounts of MLV derived vector particles bearing lacZ as a reporter gene and carrying different variants of MLV Env were attached to the fibronectin coated chamber slide. DFJ-8 cells were seeded onto the dense particle coat and β-galactosidase activity was determined by X-gal staining after 48 hours of incubation (Figure 2A). Glass bound MLV particles retained their capacity to infect DFJ-8 cells using this experimental setup. Comparison of vector particles carrying different Env proteins revealed no significant impact on transduction efficiency of the YFP or mCherry label fused to Env (Figure 2A and 2B), which is in agreement with data from Sherer and colleagues [16]. As a control, we prepared MLV-based vector particles whose fusion capabilities were impaired by a histidine-to-arginine change at position 8 (H8R) within the YFP tagged envelope protein (referred to as Env.YFP.H8R). This mutation has been shown previously to block infection by arresting virus-cell fusion at the hemifusion state [17]. As indicated in Figure 2B, the H8R mutation reduced transduction efficiency compared to wild-type by a factor of eight, while particles lacking Env did not lead to detectable transduction.

We compared the infection efficiency of immobilized particles with that of free particles to determine whether adherence to the cover slip affected the capacity of pseudotyped particles to infect DFJ-8 cells. Parallel infections were performed in which either particles or DFJ-8 cells were pre-bound to fibronectin-coated cover slips and cells or viruses were seeded on top. Infected cells were subsequently quantified by staining for β-galactosidase activity and infectivity was normalized to the particle input determined by measuring the reverse transcriptase activity of immobilized and free particles, respectively. These experiments revealed that the infectivity of the immobilized particles was equal or slightly better than that of the free particles (data not shown).

**Figure 3:**
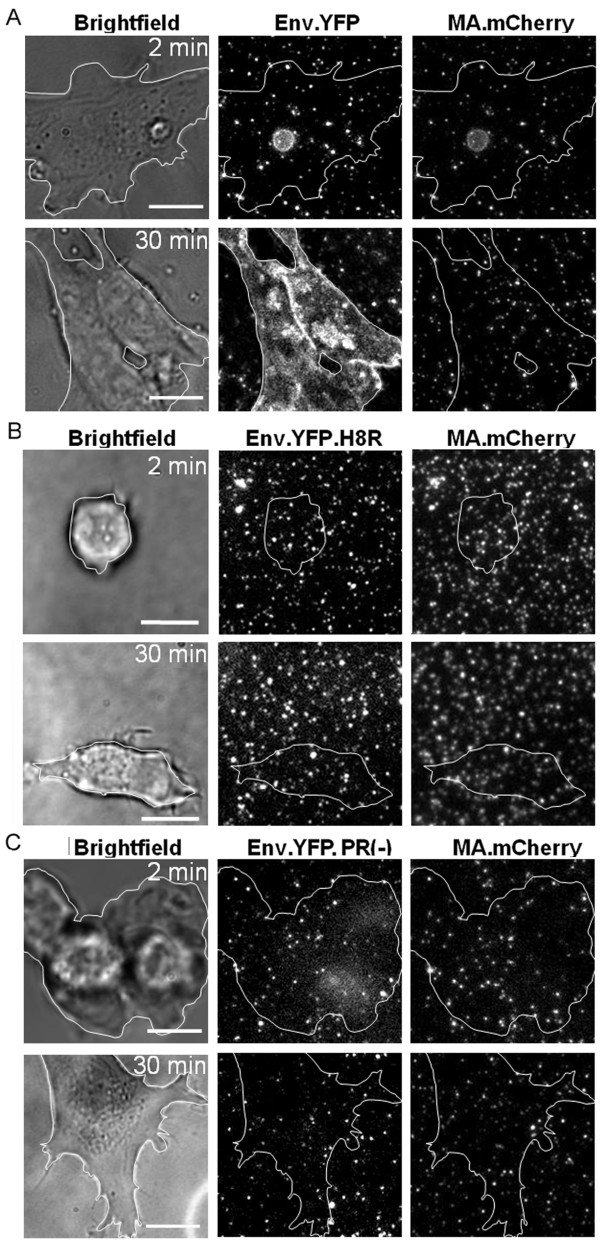
**Membrane staining of cells resulting from fusion with fluorescently labeled VLPs**. DFJ-8 cells were incubated on chambered coverslips coated with VLPs (corresponding to 500 ng p24) labeled with MA.mCherry carrying the indicated Env derivatives: (A) Env.YFP; (B) Env.YFP.H8R; (C) Env.YFP.PR(-). Cells were fixed 2 and 30 minutes after virus-cell contact, respectively, and z-stacks were recorded. Maximum projections of deconvolved z-series are shown. White lines indicate the outline of the cell as determined by bright-field microscopy. Scale bars correspond to 10 μm.

Next, we determined whether virus-cell fusion can be monitored by fluorescence microscopy using our experimental setup. Double labeled pseudotyped HIV-1 particles carrying MA.mCherry and Env.YFP were bound to fibronectin coated cover glasses and incubated with DFJ-8 cells. After 2 and 30 minutes, respectively, cells were fixed and images were recorded by performing z-stack series through the adhered cells (Figure 3). It was described previously that Env.YFP is transferred to the plasma membrane of the host cell upon fusion [16]. This was also observed for the Env.YFP pseudotyped HIV particles whose incubation with target cells led to a gradually increasing diffuse YFP staining of the plasma membrane (Figure 3A). Transfer of Env.YFP into the target cell membrane was fusion dependent and was not detected for the fusion impaired particles harboring the H8R mutation (Env.YFP.H8R; Figure 3B). Thirty minutes after cell settling, a punctate YFP and mCherry signal was seen at the cell surface, but neither a YFP nor a mCherry membrane stain was detectable (Figure 3B). As another control, double labeled particles deficient in the viral protease were used. These particles are fusion-defective because cleavage of the R-peptide from the MLV Env protein by the viral protease is necessary to render Env fusion-competent. By using a cell-cell fusion assay, particles bearing Env.YFP and deficient in protease (referred to as Env.YFP.PR(-)) were at least tenfold less fusion-competent than Env.YFP (data not shown). No significant membrane staining was detectable when cells were incubated for 30 minutes with these particles (Figure 3C). Furthermore, no Env.YFP membrane staining was detected when eMLV receptor deficient parental DF-1 cells were used instead of DFJ-8 cells (data not shown). Taken together, our results indicate that the chosen setup is appropriate for investigating viral fusion at the cell membrane by live cell microscopy.

### Visualization of individual fusion events by single particle tracing

**Figure 4:**
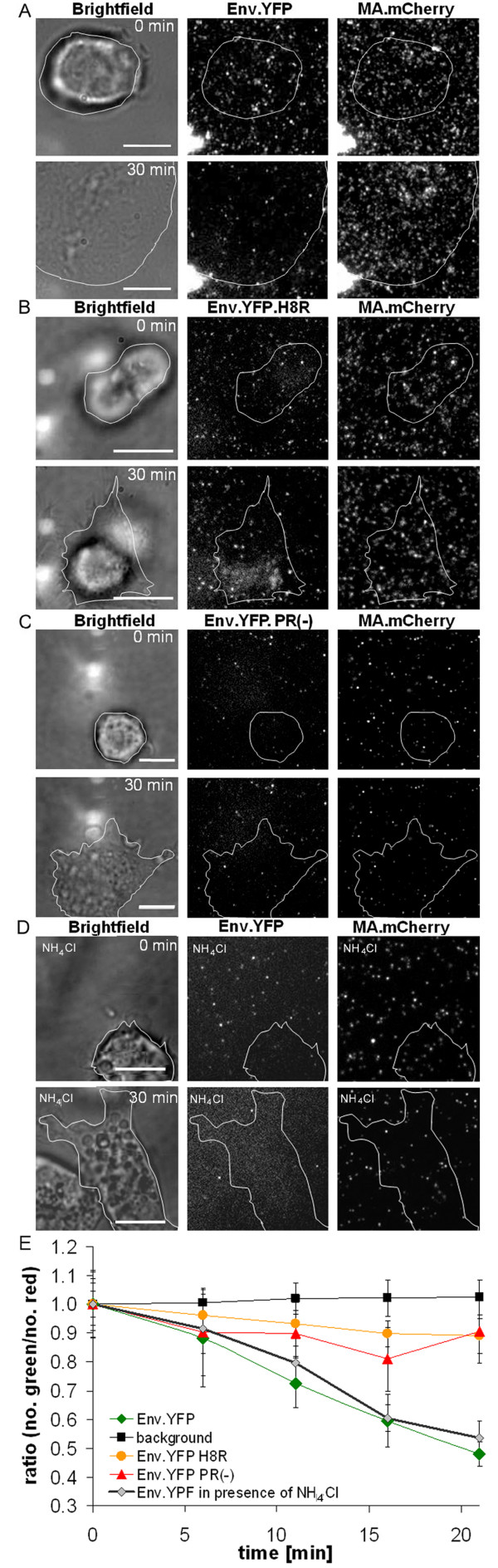
**Relative loss of the Env signal in the particle population induced by cell contact**. VLPs labeled with Env.YFP and MA.mCherry were bound to fibronectin coated chambered coverslips and incubated under live cell imaging conditions at 37°C. Particles bound to the cover slip were visualized by epifluorescence microscopy. DFJ-8 cells were added, and the moment of attachment of the cells to the coverslip was defined as time point 0. Incubation was continued at 37°C, and images were recorded at 1 frame/min. Please note that due to the experimental setup only single slices within the focal plane are depicted. (A) shows individual images of a cell on a VLP layer carrying Env.YFP recorded at the indicated time points. The outline of the cell as determined by bright field microscopy is indicated in white. Note that for both time points the same cell is shown, but the cellular morphology is changing in the early phase of attachment. (B) shows a control experiment, using fusion impaired VLPs double labeled with Env.YFP.H8R and MA.mCherry. (C) shows a control experiment, using double labeled VLPs deficient in the viral protease. (D) shows a control experiment using the same double labeled VLPs as in (A) in the presence of 30 mM NH_4_Cl. Scale bars in all depicted images correspond to 10 μm. (E) Color separation of double labeled particles over time. Images recorded at the indicated time points were evaluated using an automated tracking software. The number of red and green punctuated signals, originating from MA.mCherry and YFP-labeled Env, respectively, were determined for at least 400 single particles in three independent experiments, and the total number of red and green signals per image was quantified. The plot shows the ratio between the number of green and red signals determined as a measure for the bulk amount of double labeled particles. Quantification in regions covered by cells is shown for particles carrying Env.YFP in the absence (green) and presence of NH_4_Cl (grey), for particles carrying Env.YFP.H8R (orange) and for Env.YFP.PR(-) particles (red), respectively. As control, the same quantitative analysis was performed for the background signal of particles in areas where no cells had settled (black).

After monitoring overall virus-cell fusion by fluorescence microscopy, we were next interested in visualizing and characterizing single particle fusion events at the plasma membrane. To this end, Env.YFP and MA.mCherry double labeled particles were again immobilized on fibronectin coated cover glasses, and DFJ-8 cells were allowed to settle onto the virus like particle (VLP) coat. Image acquisition was started immediately after cell attachment to the glass bottom (defined as time point 0, Figure 4). Time resolved epifluorescence microscopy revealed a continuous reduction in the number of YFP signals originating from single virions, indicating viral fusion at the cell membrane. The number of YFP-labeled particles in areas of the cover glass where no cell had settled remained, on the other hand, largely unchanged (Figure 4A). A time series of images following settling of a cell onto the particle coat revealed a gradually appearing diffuse membrane stain (see Additional file 1, 2 and 3), indicating the cumulative effect of multiple individual fusion events. Interestingly, the signal corresponding to the labeled MA protein was not lost concomitantly with the Env.YFP signal, and a punctate pattern of mCherry on the cell surface remained even after 30 minutes of incubation (Figure 4A). Only a faint diffuse YFP membrane stain was observed for Env.YFP.H8R bearing particles upon prolonged incubation (30 minutes) and the punctate Env.YFP signal remained largely unchanged, indicating that many fewer particles had fused with the plasma membrane (Figure 4B). There was also no significant change in the MA.mCherry signal (Figure 4B). The same was observed for protease-defective particles (Figure 4C).

Quantification of the red and green signal intensities originating from MA.mCherry and Env.YFP, respectively, of at least 400 individual double labeled particles as a function of time revealed a significant loss of the Env-associated YFP signal relative to the MA-associated mCherry signal for particles bearing fusion-competent Env.YFP (approximately 50% decrease after 20 minutes) as depicted in Figure 4E. To determine whether loss of the Env-YFP signal could be due to quenching of the pH-sensitive fluorophore YFP upon exposure of endocytosed particles to the low pH of the endosome, experiments were performed in the presence of ammonium chloride which prevents endosomal acidification (Figure 4D). As indicated in Figure 4E, ammonium chloride treatment had no significant impact on the loss of the Env.YFP signal over time. Furthermore, specific loss of the Env-associated signal could also be observed when Env was labeled with the less pH-sensitive protein mCherry (data not shown). Immobilized particles which had no cell contact did not display a significant loss of the Env.YFP signal, which indicates that photobleaching also did not contribute significantly to the loss of YFP fluorescence (indicated as background in Figure 4E). As expected, fusion impaired particles (Env.YFP.PR(-) and Env.YFP.H8R bearing VLPs, respectively) showed only a minor reduction of the YFP signal (approximately 10% decrease in the first 20 minutes after cell contact).

The observation of a persistent MA signal after loss of the viral membrane was not expected considering current models of retroviral entry. To determine whether the MA shell could have been artificially stabilized by fusion of the fluorescent protein, we analyzed MA shell dissociation *in vitro *using two different approaches. First, the Env.YFP/MA.mCherry labeled particles were adhered to a glass cover slip, incubated with 0.05% Triton X-100 and the number of single and double labeled particles was recorded over time. These experiments showed a rapid and concomitant loss of both signals upon detergent addition (Additional file 4A). Second, we made use of a FRET based assay to monitor the time course of MA shell dissociation. Purified particles labeled with a mixture of MA.eCFP and MA.eYFP displayed a strong FRET signal which rapidly disappeared upon disruption of the particle membrane with 0.05% Triton X-100. As expected, stabilization of the Gag shell by prevention of Gag processing prevented the decay of this FRET signal. Dissociation of the mature MA.XFP shell (indicated by a fluorescence spectrum resembling that of free eCFP) was complete within ~10 seconds at 37°C (Additional file 4B).

**Figure 5:**
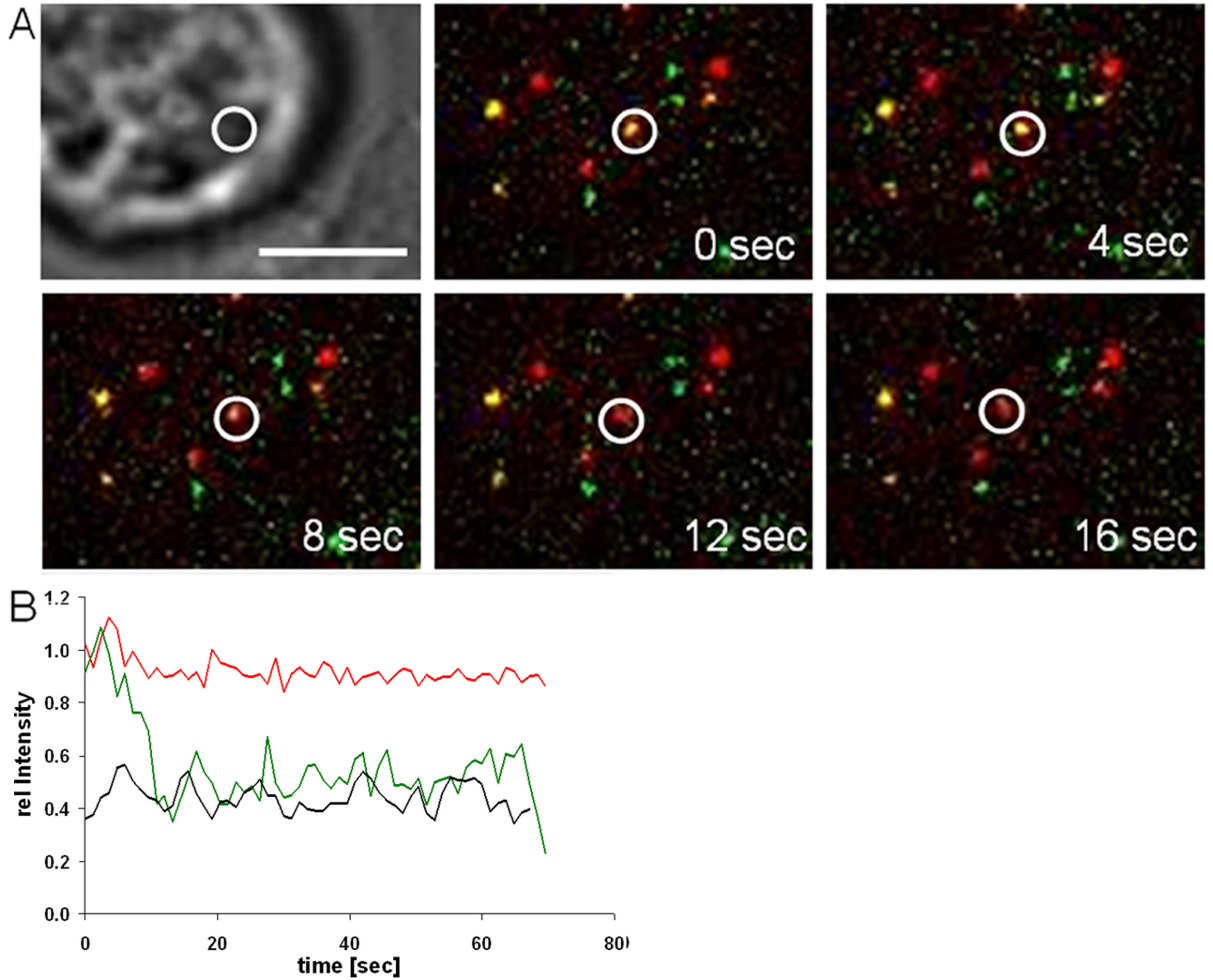
**Visualization of a fusion event in real time**. (A) MA.mCherry and Env.YFP double labeled particles were immobilized onto a fibronectin coated cover slip, and DFJ-8 cells were allowed to settle on the particle layer. Image acquisition with a frame rate of 0.76 frames/sec was started as soon as the first cells reached the microscope slide (~1 minute after cell addition; see Additional files 5 and 6). Still images taken from the movie shown in Additional file 5 at the indicated time points after the start of image acquisition are shown. The particle of interest is indicated by a white circle. Scale bar = 10 μm. (B) Plots of fluorescence as a function of time. Depicted are normalized intensity values of the Env.YFP signal (green) and the MA.mCherry signal (red) of the virus particle monitored in (A) (indicated by a white circle) and the background intensities of the Env.YFP channel (grey). Time indicates the duration of virus-cell contact in seconds.

After validating the experimental setup under bulk conditions, we proceeded to monitor single fusion events in real time. Immediately after DFJ-8 cells had contacted the layer of immobilized double labeled particles, imaging was initiated at 1 frame/second in each channel. The Additional files 5 and 6 show a time course of the initial events after virus-cell contact. Figure 5A depicts representative still images of the movie shown in Additional file 5. The white circle in Figure 5A identifies a double labeled particle which rapidly lost its Env.YFP fluorescence within the first 12 seconds after cell contact, while the MA.mCherry intensity remains unaltered, manifested by a change in particle color from yellow to red (Figure 5A). We developed an automated tracking approach to obtain quantitative data on a large number of individual virus-cell contacts that was adapted to monitor fluorescence intensities of individual particles in two channels at low signal-to-noise ratio [18]. Figure 5B shows changes in signal intensities over time for the particle indicated in Figure 5A. To acquire a statistically relevant data set, we tracked more than 20,000 individual double labeled particles. As summarized in Table 1, 28 color separation events indicating fusion were identified in the case of Env.YFP carrying particles, whereas no color separation was detected when more than 11,000 particles bearing the fusion impaired Env.YFP.H8R mutant were tracked. In 13 of those 28 events, mobility of the particle precluded continued observation of the MA signal. From the remaining 15 events, 10 resulted in a stable punctate MA signal over the remaining observation period. Examples of individual trajectories of fusion events are shown in the Additional file 7. Interestingly, 45 events of simultaneous loss of both colors were detected in the case of VLPs harboring Env.YFP, while only twelve such events were observed for particles bearing the fusion defective Env.YFP.H8R mutant.

**Table 1:** Summary of the automated tracking results.

	**Env.YFP**	**Env.YFP.H8R**
Tracks (total)	21054	11609

Fusions events	28	0

Simultaneous loss of both colors	45	12

The table represents the total number of all particles tracked by an automated tracking software [18], the number of monitored fusion events and the number of particles, where both colors were lost simultaneously. Only particles bearing Env.YFP and Env.YFP.H8R as a fusion defective control have been analyzed.

## Discussion

This study aimed at monitoring individual fusion events of eMLV Env pseudotyped HIV-1 particles and at analyzing the subsequent fate of the sub-membrane MA layer. So far, the dynamics of virus-cell fusion has been predominantly studied using cell-cell fusion assays in which cells expressing a viral Env protein fuse with cells expressing the cellular receptor for the virus [19-21]. However, the stoichiometry of Env and receptor as well as the geometry of the fusion area between two similarly sized cells do not accurately reflect the events occurring in the fusion between a small virion and a much larger cell. Analysis of cell-cell fusion events revealed an average half-time of 10 to 20 minutes [22,23]. Scoring for loss of fluorescent Env molecules from double labeled HIV/eMLV pseudotypes, 28 fusion events were identified in the present study; and individual fusion events were already observed within seconds after the first virus-cell contact. This result is in agreement with a previous study, in which fusion of individual HIV-1 Env pseudotyped viruses labeled with the lipophilic dye DiD and GFP attached to the NC domain of Gag was monitored after binding to target cells at low temperature. These authors also observed initial fusion events within the first minute after shifting the temperature to 37°C [6], and they concluded that virus-cell fusion proceeds without significant delay during rising temperature. Thus, virus-cell fusion appears to be kinetically different from cell-cell fusion.

Our approach involved pseudotyping of fluorescent HIV-1 particles carrying a fluorophor in the MA domain of Gag with fluorescent eMLV Env. Both modifications have been shown to be compatible with particle formation and infectivity [10]. Env is a membrane-embedded glycoprotein that is expected to remain attached to the plasma membrane after fusion. Accordingly, progressive plasma membrane labeling was observed upon incubation of DFJ-8 target cells with particles carrying wild-type Env, but not with particles carrying fusion-impaired or -defective variants. MA is associated with the inner leaflet of the virion membrane and is generally believed to remain at the plasma membrane after fusion before dissociating into the cytosol. Thus, the combination chosen in this report would not appear to be optimal for detecting color separation upon fusion. However, previous studies had shown bulk separation of labeled MA and inner core proteins over time when double labeled particles were incubated with permissive cells, while individual events of color separation were not detected [15]. These observations raised the possibility that HIV-1 MA may remain attached with the entering viral core for at least a short period after membrane fusion. Consistent with this hypothesis, particulate MA signals were largely retained upon incubation of target cells with immobilized double labeled particles, while the Env.YFP signal was gradually lost over time. Tracking individual double labeled particles identified 28 events of color separation, indicating that the MA layer can dissociate from the surface glycoproteins upon membrane fusion. It may then remain associated with the entering viral core, at least for a short time. 10 of the 15 particles underwent a color separation event in the live cell experiments and could subsequently be followed until the end of the data acquisition. Consistent with our hypothesis, the 10 particles displayed a punctate MA.mCherry signal over the remaining observation period (corresponding to up to 4 minutes after color separation). While this does not clearly exclude a dissociation of the punctate MA.mCherry signal at later time points, it suggests that the MA shell may at least be transiently stable after the envelope is lost. Preliminary results on triple labeled particles carrying different fluorophors in Env, MA and the viral core also support this conclusion, revealing transient co-localization of MA and the entering core after fusion-dependent loss of the Env layer (unpublished observation). These events were rare, and it is currently not clear whether they give rise to productive entry. MLV pseudotypes efficiently fuse with DFJ-8 cells, however; and they exhibit a high infectivity on these cells, making it likely that at least some of the observed events represent productive fusion. Conceivably, the observed color separation events may constitute only a minority of all fusion events with the majority not being scored because of concomitant loss of MA together with Env fluorescence. This appears unlikely, however, because only 45 further events of particles losing the fluorescent signal were detected. In these cases both colors were lost simultaneously. Concomitant disappearance of both colors could be due to loss of the particle from the focus plane (e.g. during endosomal uptake), which may explain why such events were also seen for particles pseudotyped with fusion-defective Env. The number of events was much lower in this case (12 versus 45), indicating that at least some of the observed events of simultaneous loss of both colors also represent membrane fusion. Based on this study, such events do not appear to be more common than separation of Env and MA, however.

MA carries the plasma membrane trafficking moiety of Gag and is thus responsible for Gag membrane association in the assembly phase [24]. This is mediated by N-terminal myristoylation, basic charges and a phosphatidylinositol 4,5-bisphosphate binding site in MA [25,26]. Membrane binding affinity is much lower for the cleaved MA domain than for full-length Gag [27,28]. This is due to a myristoyl switch regulating exposure of the acyl chain and due also to the lack of stable multimerisation of MA [29,30]. Accordingly, MA is rapidly stripped from the viral core upon detergent treatment [31-33], and only small amounts of MA have been detected in HIV pre-integration complexes [11,12]. The bulk of MA can thus be expected to dissociate from the membrane into the cytoplasm as monomers or small oligomers after fusion. Such redistribution of MA is in agreement with previous observations using MA.eGFP/Vpr.mCherry labeled particles. After prolonged incubation, a diffuse cytoplasmic distribution was observed for the MA.eGFP signal in this case [15]. This redistribution does not always occur directly upon fusion, however, since particulate MA.mCherry signals could be tracked for up to several minutes after loss of the Env signal in the present study. The simplest explanation for this phenotype would be the retention of a stable MA lattice at the fusion site with concomitant dissipation of Env molecules within the plasma membrane. There is currently no evidence, however, for a stable MA lattice. This hypothesis cannot explain the occasionally observed rapid movement of MA clusters after loss of the Env signal. Nor would this hypothesis be compatible with the temporary co-localisation of MA and the core in triple labeled particles. Such co-localisation could be due to a delayed opening of the fusion pore that allows dissipation of Env proteins within the plasma membrane while the core is still retained in the particle neck. A delayed release of an aqueous marker was observed after hemifusion had occurred in a previous study [6], and this could also apply to the later stages of fusion pore opening. Alternatively, the MA layer may dissociate from the membrane and remain transiently associated with the viral core after fusion and separation from the membrane. Furthermore, interaction of MA with the cytoplasmic tail of its cognate Env protein may be important for regular uncoating. Future live cell microscopy studies using high time resolution and fluorophors in different viral proteins will shed light on these immediate post-fusion events which are largely unexplored for most viruses.

## Methods

### Plasmids

The plasmid Friend MLV Env-YFP [16] was provided by W. Mothes (Yale University School of Medicine). The plasmids pMMP-LTR-LacZ and pMDoldGag-Pol were provided by Richard Mulligan (Department of Genetics, Harvard University). The plasmid 1765-H8R [17] that expresses the MLV envelope protein bearing a histidine to arginine mutation at position 8 was a gift from L. Albritton (University of Tennessee). To introduce the H8R mutation into Env.YFP we performed site directed mutagenesis using the Stratagene quick exchange kit (forward primer: 5'-CTCAGTGGGCCGCCCGATTGGGGGCTAGAGTATC-3'; reverse primer: 5'-GATACTCTAGCCCCCAATCGGGCGGCCCACTGAG-3') resulting in the plasmid Env.YFP.H8R. Plasmid pCHIV and derivatives have been described previously [15]. The plasmid pCHIV.Env(-).PR(-) carrying a point mutation in the PR active site and a frameshift mutation in the *env *gene was constructed by exchange of an *Age*I-*Xho*I fragment of pCHIV.PR(-) with the corresponding fragment of pCHIV.Env(-).Env.YFP with an uncleaved R-peptide is referred to as Env.YFP.PR(-).

### Tissue culture and production of fluorescently labeled virus particles

293T, DF-1 and DFJ-8 cells were cultured in Dulbecco's modified Eagle's medium (DMEM; Invitrogen), supplemented with 10% fetal calf serum (FCS; Biochrom), penicillin (100 IU/mL) and streptomycin (100 μg/mL). Live cell imaging studies were performed in PBS supplemented with 1 mM CaCl_2_, 0.5 mM MgCl_2 _and 1% FCS. For production of double fluorescently labeled particles, 293T cells were co-transfected with a mixture of pCHIV.Env(-), pCHIV.mCherry.Env(-), or their protease deficient variants, respectively, and the plasmid Env.YFP in a molar ratio of 1:1:4 by calcium phosphate precipitation. Supernatants were harvested at 36 hours post transfection and filtered through a 0.45 μm filter. Particles were concentrated by ultracentrifugation through a 20% (w/w) sucrose cushion. Virions were resuspended at 3 μl/ml culture supernatant in phosphate-buffered saline (PBS) supplemented with 10% FCS and 10 mM HEPES pH 7.3, frozen in liquid nitrogen, and stored at -80°C. Particle yield was determined by ELISA quantitation of the p24 capsid protein using an in house ELISA. For Western blotting, samples were separated by SDS-PAGE (16% acrylamide gels) and transferred by semi-dry blotting to an activated PVDF membrane (Immobilon-FL, Millipore). Viral proteins were detected by using polyclonal rabbit antiserum raised against recombinant HIV-1 CA protein or goat anti-Rauscher murine leukemia virus gp70 with known cross-reactivity to MLV Env (provided by C. Buchholz, Paul Ehrlich Institute, Langen). Rat polyclonal antiserum raised against mCherry was provided by Heinrich Leonhardt, LMU Munich. YFP was detected using rabbit polyclonal antiserum against recombinant GFP. Detection and documentation were performed with the Li-Cor Odyssey system according to the manufacturer's instructions, using the appropriate secondary antibodies provided by the manufacturer. MLV vector particles transducing β-galactosidase were quantified either by immunoblotting against the p30 CA protein (antiserum kindly provided by C. Buchholz, Paul Ehrlich Institute, Langen) or by measuring their reverse transcriptase activity using the RETRO SYS, RT Activity Kit (Innovagen AB) as recommended by the manufacturer.

### Analysis of viral infectivity

To determine viral infectivity, MLV vector particles carrying the lacZ gene and the indicated Env proteins were generated as described previously [16]. Briefly, 293T cells were co-transfected with 5 μg of a plasmid encoding the vector RNA (pMMP-LTR-LacZ), 5 μg plasmid encoding wild-type Env or its labeled derivatives, respectively, and 5 μg plasmid encoding wild-type GagPol (pMDoldGag-Pol) in a 10 cm dish by calcium phosphate precipitation. The medium was changed 24 hours and 36 hours post transfection, the medium was harvested and particles were purified as described above. Comparable amounts of particles (as determined by p30 immunoblot) were adhered to fibronectin-coated coverslips and DFJ-8 cells were allowed to settle on top of the virus coated surface. After 48 hours cells were fixed with 4% PFA and β-galactosidase activity was determined by X-gal staining. The percentage of infected cells was determined by the ratio of stained cells to total cells.

### Microscopy

Epifluorescence microscopy was performed on a Zeiss Axiovert 200 M microscope with a back illuminated EM-CCD camera (Cascade II, Roper Scientific). Images were acquired with Metamorph Software (Visitron). For live cell imaging, cells were incubated at 37°C in a microscope incubation chamber (EMBLEM, Heidelberg, Germany). The microscopic setup has been described previously [15]. For experiments analyzing single particle fusion, eight-chambered cover glasses (LabTek, Nunc) were coated with fibronectin (Sigma) at a concentration of 100 μg/μl and incubated at 37°C for 1 h. Fibronectin was removed and the cover glasses were dried for 30 minutes and rinsed with PBS. Fluorescent virus particles in PBS were subsequently added to the chambers. To detect overall changes in the VLP population, we used a VLP amount corresponding to 500 ng p24. For single event tracing, a VLP amount corresponding to 100 ng p24 was used. VLPs were allowed to adhere to fibronectin for 30 minutes at room temperature before removal of the virus containing solution. Subsequently, a suspension containing approximately 5,000 DFJ-8 cells was added in pre-warmed PBS supplemented with 1 mM CaCl_2 _and 0.5 mM MgCl_2 _and 1% FCS. Image acquisition was started when cells attached to the bottom of the cover glasses. Cell positions were documented by bright field images recorded immediately before and after the time series. To block endosomal acidification, DFJ-8 cells were trypsinized and incubated in the presence of 30 mM NH_4_Cl for 3 h at 37°C. Afterwards the cells were added to prebound VLPs in PBS containing 30 mM NH_4_Cl and image acquisition was started.

### Automated particle tracking

For automated analysis a 2D tracking approach was developed to track dual-colored particles with a low signal-to-noise ratio. Details of the particle localization and tracking algorithms are described elsewhere [18]. Briefly, particles were localized using 2D Gaussian fitting and particle positions and fluorescence intensities in both YFP and RFP channels were recorded. The particles were tracked in consecutive frames using a probabilistic scheme based on the Kalman filter. To detect color separation or events where both labels vanished simultaneously the intensity profiles of each track in both green and red channel were analyzed. For this, the intensity profiles, which were derived from the automatically generated VLP traces of both channels, were compared to the corresponding background level. the mean intensity level of both signals had to differ by at least one standard deviation from the background signal to be considered as a double labeled particle. Color separation was defined as a drop of one signal to background intensity. Supplemental method information is available in Additional file 8.

## Competing interests

The authors declare that they have no competing interests.

## Authors' contributions

PK and ML performed the experimental work. MJL, BM and HGK conceived the study and designed individual experiments. WJG and KR developed the tracking software. PK, BM, MJL and HGK wrote the manuscript. All authors read and approved the final manuscript.

## Supplementary Material

Additional file 1 **Movie of a cell settling onto a glass cover slip**. Brightfield movie of a DFJ-8 cell settling onto a glass cover slip coated with Env.YFP and MA.mCherry double labeled particles (as shown in the Additional files 2 and 3). The time series was started at the time point of cell attachment to the cover glass. Images were acquired at 1 frame per minute over a period of 20 minutes. The video is played at a speed of 10 frames per second. Click here for file

Additional file 2 **Movie illustrating the visualization of retroviral fusion indicated by the gradual appearance of a diffuse membrane stain**. Corresponding to the Additional files 1 and 3 the movie S2 shows the Env.YFP signals (green channel) of the coated Env.YFP and MA.mCherry double labeled particles coated onto a glass cover slip while DFJ-8 cells attach to the cover glass. The time series was started at the time point of cell attachment to the cover glass. Images were acquired at 1 frame per minute over a period of 20 minutes. The video is played at a speed of 10 frames per second. Click here for file

Additional file 3 **Movie illustrating the distribution of HIV-1-Matrix during retroviral fusion**. Corresponding to the Additional files 1 and 2 the movie shows the MA.mCherry signals (red channel) of the coated Env.YFP and MA.mCherry double labeled particles coated onto a glass cover slip. During the observation time DFJ-8 cells attach to the cover glass (see Additional file 1). The time series was started at the time point of cell attachment to the cover glass. Images were acquired at 1 frame per minute over a period of 20 minutes. The video is played at a speed of 10 frames per second. Click here for file

Additional file 4 **Dissociation of mature and immature particles upon detergent treatment**. (A) Time course of particle dissociation induced by detergent treatment under imaging conditions. Particles labeled with both MA.mCherry and Env.YFP were adhered to a glass coverslip and imaged with a time resolution of 1 frame/sec. At 15 sec after the start of observation (arrow) Triton-X100 was added to a final concentration of 0.05%, and observation was continued. At 20, 30, 40 and 50 sec after the start of the observation, the numbers of punctuate double labeled (yellow line) and MA.mCherry signals (red line) were quantified. As a control, double labeled PR(-) particles were subjected to the same procedure, and the punctate Gag.mCherry signal was quantified (blue line). The numbers were normalized to the values obtained at the beginning of observation (t = 0). The plot shows data from one representative experiment out of 3 independent experiments. (B) Time course of MA.XFP shell dissociation in vitro monitored by FRET analysis. Mature or immature FRET reporter particles labeled with eCFP and eYFP fused to the MA domain of Gag, were prepared as described in the supplementary methods. Fluorescence measurements were carried out at 25°C or 37°C, respectively, using an excitation wavelength of 433 nm. At t = 0, Triton-X100 was added to a final concentration of 0.05%, and fluorescence emission at 528 nm was monitored over time. Volume corrected values were normalized to the emission intensity recorded before detergent addition. Click here for file

Additional file 5 **Movie displaying an individual fusion event indicated by color separation, corresponding to still images in Figure **5A. Env.YFP (green) and MA.mCherry (red) labeled particles were coated onto a glass coverslip, and DFJ-8 cells were allowed to settle onto the virus particles. Image acquisition with a time resolution of 0.76 frames/sec was started at the time point of cell attachment to the coverslip. The video shows a section of the movie covering 38 sec and is displayed at a speed of 10 frames per second. The particle of interest is indicated by a white circle. While the Env.YFP signal vanished within 15 sec after virus-cell contact, the label of the MA domain remained punctated during the remaining period of observation. Still images of the video are shown in Figure 5A. Click here for file

Additional file 6 **Movie displaying an individual fusion event indicated by color separation, followed by disappearance of the punctate MA.mCherry signal**. Env.YFP (green) and MA.mCherry (red) labeled particles were coated onto a glass coverslip, and DFJ-8 cells were allowed to settle onto the virus particles. Image acquisition with a time resolution of 0.76 frames/sec was started when the cell contacted the coverslip. The Env.YFP signal vanished within 30 sec after virus-cell contact and the punctate MA.mCherry signal disappeared 4 sec afterwards. The video shows a section of the movie covering 35 sec and is played at a speed of 10 frames per second. The particle of interest is indicated by a circle. Click here for file

Additional file 7 **Representative trajectories of individual particles undergoing color separation**. (A-C) Trajectories of individual particles from live cell imaging data, similar to those shown in Additional files 5 and 6, were derived by automated tracking as described in Materials and Methods. White lines depict the outline of the cells as determined by bright field microscopy. Blue circles indicate the initial attachment sites of the particles at the cell surface. To visualize the color separation event, the tracks of the complete double labeled particles are presented in yellow, while the red part of the trajectories indicates the movement of mCherry labeled subviral particles following loss of the Env signal. Scale bars: 5 μm. Click here for file

Additional file 8 **Supplementary materials and methods.** Click here for file
