# Supplementary figures and images for: Visualizing fusion of pseudotyped HIV-1 particles in real time by live cell microscopy
Source: Retrovirology. 2009 Sep 18;6:84. doi: 10.1186/1742-4690-6-84 (PMC2762461; doi:10.1186/1742-4690-6-84)

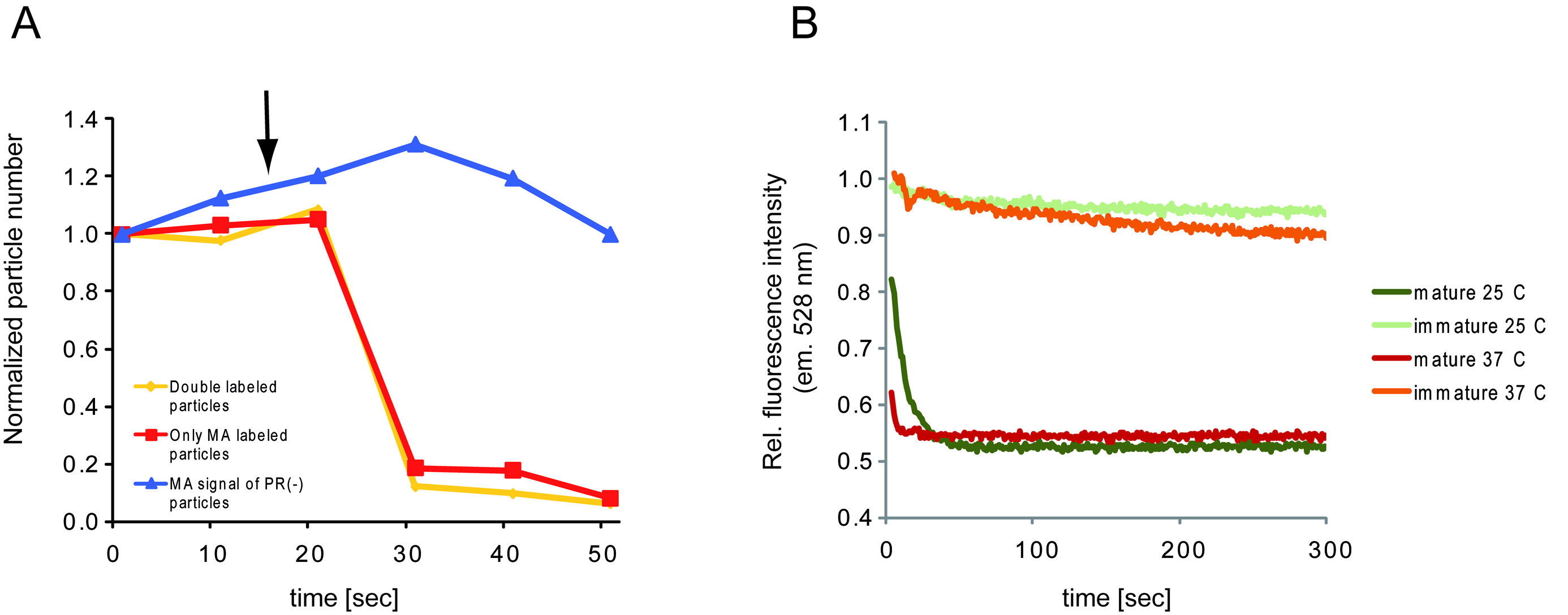

Supplement: Additional file 4 — Dissociation of mature and immature particles upon detergent treatment. (A) Time course of particle dissociation induced by detergent treatment under imaging conditions. Particles labeled with both MA.mCherry and Env.YFP were adhered to a glass coverslip and imaged with a time resolution of 1 frame/sec. At 15 sec after the start of observation (arrow) Triton-X100 was added to a final concentration of 0.05%, and observation was continued. At 20, 30, 40 and 50 sec after the start of the observation, the numbers of punctuate double labeled (yellow line) and MA.mCherry signals (red line) were quantified. As a control, double labeled PR(-) particles were subjected to the same procedure, and the punctate Gag.mCherry signal was quantified (blue line). The numbers were normalized to the values obtained at the beginning of observation (t = 0). The plot shows data from one representative experiment out of 3 independent experiments. (B) Time course of MA.XFP shell dissociation in vitro monitored by FRET analysis. Mature or immature FRET reporter particles labeled with eCFP and eYFP fused to the MA domain of Gag, were prepared as described in the supplementary methods. Fluorescence measurements were carried out at 25°C or 37°C, respectively, using an excitation wavelength of 433 nm. At t = 0, Triton-X100 was added to a final concentration of 0.05%, and fluorescence emission at 528 nm was monitored over time. Volume corrected values were normalized to the emission intensity recorded before detergent addition. [file 1742-4690-6-84-S4.tiff]

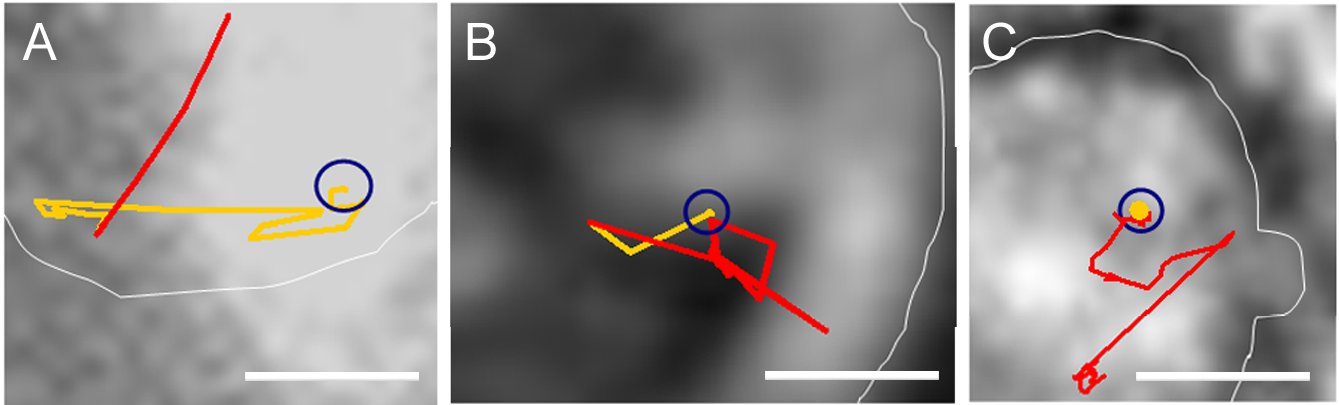

Supplement: Additional file 7 — Representative trajectories of individual particles undergoing color separation. (A-C) Trajectories of individual particles from live cell imaging data, similar to those shown in Additional files 5 and 6, were derived by automated tracking as described in Materials and Methods. White lines depict the outline of the cells as determined by bright field microscopy. Blue circles indicate the initial attachment sites of the particles at the cell surface. To visualize the color separation event, the tracks of the complete double labeled particles are presented in yellow, while the red part of the trajectories indicates the movement of mCherry labeled subviral particles following loss of the Env signal. Scale bars: 5 μm. [file 1742-4690-6-84-S7.tiff]
