# Supplementary material for: Visualizing fusion of pseudotyped HIV-1 particles in real time by live cell microscopy
Source: Retrovirology. 2009 Sep 18;6:84. doi: 10.1186/1742-4690-6-84 (PMC2762461; doi:10.1186/1742-4690-6-84)
Supplement: Additional file 8 — Supplementary materials and methods. [file 1742-4690-6-84-S8.doc]

**Supplementary materials and methods**

**FRET measurements:** For FRET measurements, reporter particles were prepared from the supernatant of 293T cells transfected with a mixture of pCHIV, pCHIVeCFP and pCHIVeYFP at a molar ratio of (2:1:1). To obtain immature particles, transfected cells were grown in the presence of the HIV-1 protease inhibitor lopinavir (f.c. 2 µM). Tissue culture supernatant was harvested at 44 h post transfection and particles were purified by ultracentrifugation through a 20 % (w/w) sucrose cushion. The particles were diluted in PBS to a concentration of approximately 10 ng CA / µl. Fluorescence measurements were carried out using an SLM AB-2 fluorescence spectrophotometer at 25 °C or 37 °C, respectively, with the excitation wavelength set to 433 nm.
